# 

**Published:** 1879-04

**Authors:** 


					﻿ESTABLISHED IN 1855.
Volume XVII.	APRIL 1879.	N UMBER
ATLANTA
MEDICAL? SURGICAL
JOURNAL.
MONTHLY: THREE DOLLARS A FEAR, JN-.iDYANCE.
EDITOR :
J. G. WESTMORELAND, M.D.,
Professor of Materia Medica in Atlanta Medv-al College.
, H. H. DICKSON, PUBLISHER.
PAX ET SC1ENTIA, SEI) VERITAS SINE TIMORE.
ATLANTA, GEORGIA:
II. II. DICKSON, ROOK AND JOD PRINTER AND BOOKBINDER.
1879.
				

